# Etiology of Ascites and Pleural Effusion Associated with Ovarian Tumors: Literature Review and Case Reports of Three Ovarian Tumors Presenting with Massive Ascites, but without Peritoneal Dissemination

**DOI:** 10.1155/2015/414019

**Published:** 2015-12-17

**Authors:** Ai Miyoshi, Takashi Miyatake, Takeya Hara, Asuka Tanaka, Naoko Komura, Shinnosuke Komiya, Serika Kanao, Masumi Takeda, Mayuko Mimura, Masaaki Nagamatsu, Takeshi Yokoi

**Affiliations:** Department of Obstetrics and Gynecology, Kaizuka City Hospital, 3-10-20 Hori Kaiduka-shi, Osaka-fu 597-0015, Japan

## Abstract

Borderline ovarian tumors are benign but relatively large tumors that are often initially mistaken as ovarian cancers. We report three cases of stage I borderline ovarian tumors having massive ascites that we (preoperatively) suspected of being advanced ovarian cancer. The three patients (35, 47, and 73 years old) reported feeling fullness of the abdomen before consulting their gynecologist. By CT scan, they were diagnosed with a pelvic tumor accompanied by massive ascites, the diameters of which were 11, 20, and 11 cm, respectively. Postsurgical pathology showed all were stage I borderline ovarian tumors without dissemination; two were mucinous and one was serous. The amount of ascites was 6,300, 2,600, and 3,600 mL, respectively, and was serous in all. Cytodiagnosis of the ascites found that one was positive for tumor cells and two were negative. After resection of the mass, the ascites disappeared in all three cases. No pleural effusion was present at any time. The literature is reviewed concerning ascites and pleural effusions linked to ovarian tumors, and a supposition is forwarded of why pleural effusion presents sporadically in these cases.

## 1. Introduction

Borderline ovarian tumors represent 10–15% of all ovarian tumors [1]. This benign malignancy is defined as an ovarian epithelial tumor with a stratification of the epithelial lining lacking of frank stromal invasion; it has a less aggressive behavior than more invasive epithelial ovarian tumors, and the prognosis for those patients with the disease limited to the ovary is excellent [2]. However, the size of borderline ovarian tumors is relatively large, so they are often suspected of being ovarian cancers, having a much direr prognosis [3].

We report here on three cases of benign stage I borderline ovarian tumors with massive ascites that, before their operation, were suspected of being advanced ovarian cancers. Their ascites disappeared rapidly after resection of the mass and, strikingly, they lacked pleural effusion. We include a review of the literature concerning conjectures as to why pleural effusions do or do not occur in cases of pelvic tumors.

## 2. Case Presentation

### 2.1. Case 1

A 35-year-old woman felt a fullness (distension) of her abdomen before consulting a gynecologist. She was diagnosed with a pelvic tumor by CT scan and was sent to our hospital for medical treatment. The maximum diameter of her pelvic tumors was 11 cm and was accompanied by massive ascites. A total abdominal hysterectomy (TAH), bilateral salpingo-oophorectomy (BSO), and a partial omentectomy (POM) were performed.

**Figure 1:**
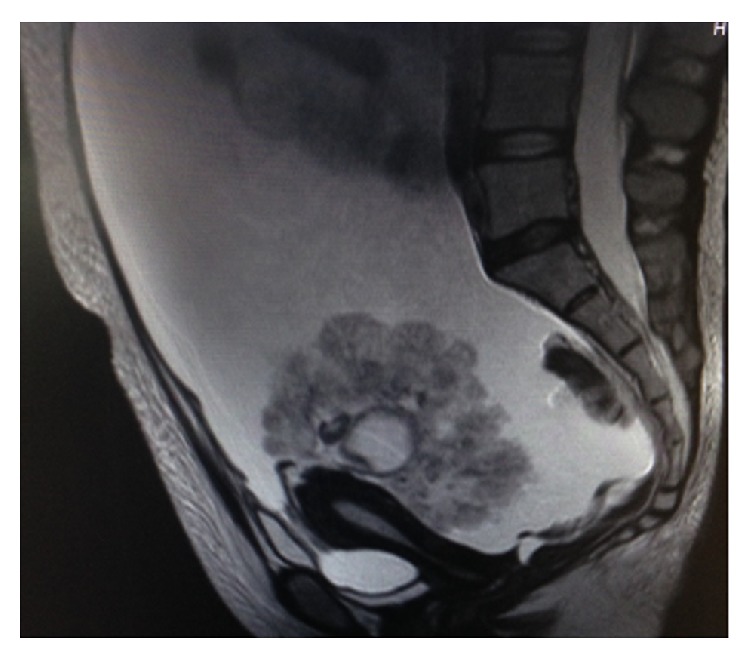
MRI image of patient 1 before operation.

**Figure 2 fig2:**
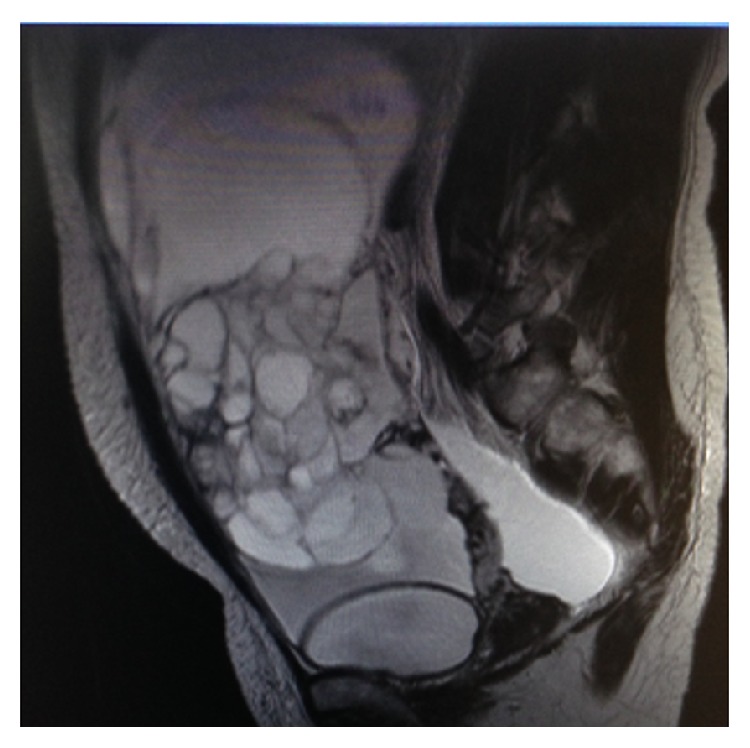
MRI image of patient 2 before operation.

**Figure 3:**
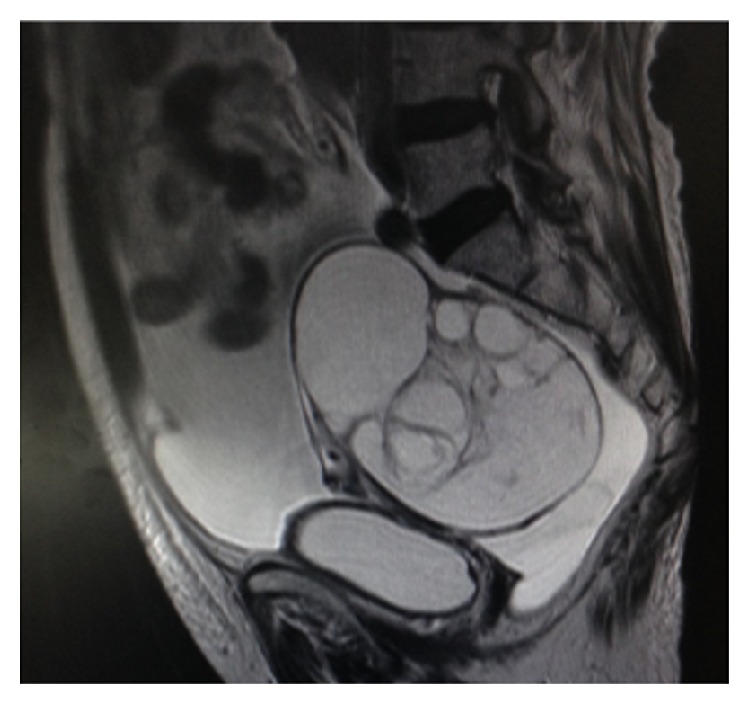
MRI image of patient 3 before operation.

Postoperatively, a pathological examination determined that the case was stage I serous borderline ovarian tumor. The amount of the serous ascites present was 6,300 mL, and the cytodiagnosis of the ascites was positive for floating tumor cells. After resection of the mass, and without adjuvant chemotherapy, the ascites disappeared rapidly. No recurrence has been found to date; the disease-free survival intervals were 1,264 days at last checkup (Table 1, Figures 1 [Fig fig2]–3).

### 2.2. Case 2

A 47-year-old woman felt a fullness (distension) of her abdomen before consulting a gynecologist. She was diagnosed with a pelvic tumor by CT scan and was sent to our hospital for medical treatment. The maximum diameter of her pelvic tumors was 20 cm and was accompanied by massive ascites. A total abdominal hysterectomy (TAH), bilateral salpingo-oophorectomy (BSO), and a partial omentectomy (POM) were performed.

Postoperatively, a pathological examination determined that the case was stage I mucinous borderline ovarian tumor. The ascites was serous. The amount of the ascites present was 2,600 mL, and the cytodiagnosis of the ascites was negative. After resection of the mass, and without adjuvant chemotherapy, the ascites disappeared rapidly. No recurrence has been found to date; the disease-free survival intervals were 1,785 days at last checkup (Table 1, Figures 1–3).

### 2.3. Case 3

A 73-year-old woman felt a fullness (distension) of her abdomen before consulting a gynecologist. She was diagnosed with a pelvic tumor by CT scan and was sent to our hospital for medical treatment. The maximum diameter of her pelvic tumors was 11 cm and was accompanied by massive ascites. A total abdominal hysterectomy (TAH) and bilateral salpingo-oophorectomy (BSO) were performed.

Postoperatively, a pathological examination determined that the case was stage I mucinous borderline ovarian tumor. The ascites was serous. The amount of the ascites present was 3,600 mL, and the cytodiagnosis of the ascites was negative. After resection of the mass, and without adjuvant chemotherapy, the ascites disappeared rapidly. No recurrence has been found to date; the disease-free survival intervals were 1,750 days at last checkup (Table 1, Figures 1–3).

## 3. Discussion

The reason often given as to why massive amounts of ascites fluid accumulate in the peritoneal cavity in cases of borderline ovarian tumors is that the fluid is the product of cul-de-sac tumor implants [4]. However, of the three cases presented, all were stage I tumors with massive ascites, yet none had metastatic implants. The ascites was serous (clear) in all three cases, so they were not due to the rupture of a mucinous ovarian tumor cyst, nor a pseudomyxoma, as occurs in some cases [5]. In the serous borderline ovarian tumor case, our cytodiagnosis of the ascites was positive for tumor cells, so the tumor may have ruptured prior to the surgery, but there was no macroscopic indication during the operation of a ruptured tumor site.

It is noteworthy that in all three cases the ascites disappeared completely after resection of the mass, even without adjuvant chemotherapy, and that there has been no recurrence of disease. This strongly suggests that the ovarian tumor had either produced or induced the ascites. This common symptom, of massive ascites without peritoneal implants, is similar to that of Meigs' and pseudo-Meigs' syndromes, although our three cases fall outside of their syndrome criteria because they lacked hydrothorax (pleural effusion).

True Meigs' syndrome is characterized by a triad of symptoms: abdominal ascites and right-sided pleural effusion associated with a benign ovarian tumor, most commonly a fibroma, but occasionally a fibroma-like Brenner or granulosa cell tumor [6, 7]. A condition is termed “pseudo-” Meigs' syndrome when it is associated with any other type of ovarian tumor, such as a mature teratoma, struma ovarii, metastatic ovarian tumor, or leiomyoma [8]. A rare “atypical” form of Meigs' syndrome can also occur; it is characterized by a benign pelvic mass with right-sided pleural effusion, but without ascites [9]. The central characteristic symptoms of these Meigs syndromes, ascites and pleural effusion, generally disappear rapidly after resection of the ovarian tumor, and, by definition, because there is no dissemination, there is no tumor recurrence [8].

The pathophysiology of ascites in ovarian tumors in general, and Meigs syndrome in particular, remains speculative. Several theories concerning the source of ascites in both Meigs' and pseudo-Meigs' syndromes have been proposed. Some involve proposed production of ascitic fluid by the tumor; others suggest that lymphatic obstruction, hormonal stimulation, release of inflammatory mediators, or tumor torsion is the cause. One early suggestion was that a solid ovarian tumor could physically irritate the peritoneum and stimulate the overproduction of peritoneal fluid [6]. Another theory proposed that the solid tumor compresses underlying lymph vessels and veins [8], slowing normal peritoneal fluid reabsorption and lymph drainage. As reviewed by Rubinstein et al., yet other theories suggested that the syndrome results from a discrepancy between the arterial supply to a large tumor mass tissue and the venous and lymphatic drainage of the same mass, leading to stromal edema and transudation [10]. Another possibility is that pressure on the lymphatics in the tumor itself may cause the escape of fluid through the superficial lymphatics of the tumor. Yet another scenario is that the ascites is caused by cytokine-induced excessive production of fluid by the peritoneum.

Release of multiple inflammatory cytokines into the plasma, including IFN*γ*, IL-1*β*, IL-6, IL-8, IL-10, TNF*α*, PlGF, and HSP90B1, is frequently associated with epithelial ovarian cancers (EOC) [11, 12]. These cytokines can be released by tumor cells, supporting stromal cells or reactive immune cells, and could play cumulative roles in the formation of ascites [12]. The hypersecretion of vascular endothelial growth factor (VEGF) from the oviducts has been singled out to play a role in the pathogenesis of ascites [13].

The etiologies of ascites and pleural effusions of the Meigs spectrum of syndromes are equally poorly understood. The example of “atypical” Meigs' syndrome with “pleural effusion without associated ascites” suggests that the two body cavity fluid pools can be, in rare cases, independent pathological conditions.

It is known that pleural effusions can result from inflammation, such as in infection or autoimmune disease, or from several other fairly common conditions. However, the pleural effusion associated with the hydrothorax in Meigs' and pseudo-Meigs' syndrome is thought to be caused by migration of excessive ascites fluid into the pleural cavity via a unique class of lymphatic channels in the diaphragm [14, 15]. The mechanism of pleural effusion development is even more obscure when in the absence of ascites, as in some cases of atypical Meigs. The pleural effusion is classically, though not exclusively, a transudate. It has been proposed that because the transdiaphragmatic lymphatic channels are larger in diameter on the right, the pleural effusion in Meigs syndromes is classically on the right side too. However, left-sided-only and bilateral pleural effusions have been reported [16].

In Meigs' and pseudo-Meigs' syndrome, the amount of pleural effusion that accumulates usually has no direct relationship with the amount of ascites present [17], nor is there a particular kind of ovarian tumor or type of ascites predisposed towards producing Meigs' and pseudo-Meigs' syndrome's characteristic pleural effusion [17]. There have been enumerable cases of each tumor type, accompanied by massive serous ascites, where a pleural effusion was absent.

Presumably, whether hydrothorax occurs as a consequence of ascites depends on the presence and nature of a pathway for ascites flow from the abdomen to the pleural cavity. It is thus illustrative to note that a known complication of peritoneal dialysis for renal disease is the movement of the dialysate into the pleural space, causing a serous hydrothorax [18]. Peritoneal dialysis-related hydrothorax is almost uniformly right-sided and represents one of many presentations of the “porous diaphragm syndrome.”

In addition to diaphragm porosity, the inherent intestinal circulation, lower hydrostatic pressure in the right upper quadrant, and liver capsule may contribute to this right-sided predominance. In addition to Meigs syndrome, similar right-sided presentations have been described in bilious effusions with gastric or duodenal perforations, hepatic hydrothorax, and nephrotic syndrome-related chylothorax [17]. Because peritoneal dialysis-induced hydrothorax is relatively rare in this commonly done procedure, it suggests that there are individual differences in susceptibility to the condition.

LeVeen et al. reported that hydrothorax occurred in only 5.3 percent of their ascitic patients. Eighteen of the 21 spontaneous hydrothorax cases in their study occurred on the right side [19]. They proposed that the pleural effusion is derived from ascitic fluid that enters the chest because of the negative pressure within the pleural space, and it does so via tiny defects in the diaphragm. Studies have shown that these tiny defects are often covered by pleuroperitoneum, but the high abdominal pressure accompanying ascites accumulation raises a bleb on the superior surface of the diaphragm. Rupture of the blebs produces the hydrothorax. The broken bleb flap acts as a one-way valve. The ascites is often initially relieved with the onset of the hydrothorax [19]. A direct thoracoabdominal communication was confirmed by a scan of the chest and abdomen after intraperitoneal injection of contrast material. Peritoneal-to-pleural flow of fluid was demonstrated by nuclear scanning, even when ascites was not clinically apparent [20].

We now know much more about the microscopic process of transdiaphragmatic fluid flow. Lymphatics in the diaphragm form a specialized system for draining fluid from the peritoneal cavity and returning it to the vascular system [21]. Fluid from the abdomen enters subperitoneal lymphatic lacunae located between muscle fibers of the diaphragm. The lacunae are separated from the peritoneal cavity by a barrier comprising, successively, lymphatic endothelium, a layer of collagenous fibers, a thin fenestrated layer of elastic tissue, and the peritoneal mesothelium. To reach the lacunae, peritoneal fluid passes through specialized stomata located between cuboidal mesothelial cells of the lacunar roof. Stoma patency is modifiable in response to stretch (as from accumulating ascites) or active induction. Nitric oxide (NO), often associated with inflammatory cells, enlarges the lymphatic stomata to increase the peritoneal lymph drainage [22].

While the distribution of mesothelial stomata across abdominal organ surfaces, and subjacent lymphatic lacunae, varies in different species, these stomata appear to be predominantly on the peritoneal surface of the diaphragm. From the lacunae, fluid traverses the diaphragm via intrinsic lymphatic channels to reach collecting lymphatics beneath the diaphragmatic pleura. Both intrinsic and collecting lymphatics contain valves [21]. These one-way channels serve as the main drainage route for absorption of ascites from the peritoneal cavity into the lymph system for return to the blood vascular system. The collecting lymphatics of the diaphragm drain principally into retrosternal (parasternal) lymphatic trunks that carry lymph to the great veins after it filters through mediastinal lymph nodes.

Clinically, these channels provide escape for tumour cells, pathogens, and toxins from the peritoneal cavity. They can also provide access for blood transfusions, for intraperitoneal chemotherapy to treat malignancies, and for peritoneal dialysis in treating chronic renal failure.

Based on the most likely of these conjectures, and on scientific findings, we suggest that Meigs-associated hydrothorax is the result of the existence of either more numerous or more permissive lymphatic channels in the diaphragm of predisposed individuals. It follows that there can be bilateral differences in the distribution of these channels, and differences among individuals for pleural effusion. The corollary is that there may be individuals who have minimal numbers of lymphatic channels in the diaphragm and are thus more resistant to hydrothorax and more predisposed to ascites accumulation. In such cases, pleural effusion would be far less likely to occur, despite the existence of massive ascites.

In all our cases, no one wished to preserve their reproductive capacity. But one-third of borderline ovarian tumors occur in women younger than 40 years, so they often wish that [23]. Ludovisi et al. reported the ultrasonographic findings that are helpful in diagnosis of serous ovarian tumors and serous surface papillary borderline ovarian tumors. Given these findings, it is possible to perform fertility-sparing surgery in those cases [24].

In conclusion, in our normal practice we have encountered three cases of borderline ovarian tumor accompanied with massive ascites. They were atypical in that they were not advanced ovarian cancers, nor did they fit within the criteria of Meigs' or pseudo-Meigs' syndrome, because they lacked pleural effusion. This is not unusual in that various types of benign to highly malignant ovarian tumors may present as massive ascites of unknown origin.

Physicians should be aware of the differential diagnostics needed to determine what type of mass they are dealing with. Pathological confirmation of the tumor and its surgical removal are primary goals in such cases, in order to avoid unnecessary neoadjuvant therapy and radiation/chemotherapy.

## Figures and Tables

**Table 1:** The list of the patients.

	Patient 1	Patient 2	Patient 3
Age (y.o.)	35	47	73

GP	G0	G2P2	G2P2

Diameter of the tumor (cm)	11	20	11

Tumor markers (U/mL)	CA125: 715.6 CA19-9: 23	CA125: 176 CA19-9: 23	CA125: 103.1 CA19-9: 250

Operation	AT + BSO + POM	AT + BSO + POM	AT + BSO

Amount of ascites (mL)	6300	2600	3600

Cytology of ascites	Positive	Negative	Negative

Pathology	Serous borderline tumor	Mucinous borderline tumor	Mucinous borderline tumor

Stage	Ic pT1cNXM0	Ic pT1cNXM0	Ia pT1aNXM0

Disease-free survival (days)	1264	1785	1750
